# M2e-Displaying Virus-Like Particles with Associated RNA Promote T Helper 1 Type Adaptive Immunity against Influenza A

**DOI:** 10.1371/journal.pone.0059081

**Published:** 2013-03-18

**Authors:** Lorena Itatí Ibañez, Kenny Roose, Marina De Filette, Michael Schotsaert, Jessica De Sloovere, Stefan Roels, Charlotte Pollard, Bert Schepens, Johan Grooten, Walter Fiers, Xavier Saelens

**Affiliations:** 1 Department for Molecular Biomedical Research, VIB, Ghent, Belgium; 2 Department of Biomedical Molecular Biology, Ghent University, Ghent, Belgium; 3 Unit of Surveillance, Orientation and Veterinary Support, Operational Direction Interactions and Surveillance, Veterinary and Agrochemical Research Centre (CODA/CERVA), Brussels, Belgium; 4 Virology Unit, Department of Biomedical Sciences, Institute of Tropical Medicine, Antwerp, Belgium; University of Edinburgh, United Kingdom

## Abstract

The ectodomain of influenza A matrix protein 2 (M2e) is a candidate for a universal influenza A vaccine. We used recombinant Hepatitis B core antigen to produce virus-like particles presenting M2e (M2e-VLPs). We produced the VLPs with and without entrapped nucleic acids and compared their immunogenicity and protective efficacy. Immunization of BALB/c mice with M2e-VLPs containing nucleic acids induced a stronger, Th1-biased antibody response compared to particles lacking nucleic acids. The former also induced a stronger M2e-specific CD4^+^ T cell response, as determined by ELISPOT. Mice vaccinated with alum-adjuvanted M2e-VLPs containing the nucleic acid-binding domain were better protected against influenza A virus challenge than mice vaccinated with similar particles lacking this domain, as deduced from the loss in body weight following challenge with X47 (H3N2) or PR/8 virus. Challenge of mice that had been immunized with M2e-VLPs with or without nucleic acids displayed significantly lower mortality, morbidity and lung virus titers than control-immunized groups. We conclude that nucleic acids present in M2e-VLPs correlate with improved immune protection.

## Introduction

Human influenza is a highly contagious respiratory disease caused by influenza A and B viruses, which undergo frequent antigenic changes. Few infections are as detrimental as influenza in terms of school and work absenteeism, medical consultation load, hospitalization, and death toll [1]. Seasonal influenza is currently counteracted by vaccination with a tri- or quadrivalent vaccine based on the hemagglutinin antigen, which is highly variable. Therefore, the composition of the influenza vaccine is adapted nearly every year so that it corresponds as much as possible to the antigenicity of the strains expected to circulate during the upcoming season [2]. The World Health Organization conducts continuous global surveillance of new influenza virus strains and guides the annual updating of the vaccines [3].

Human influenza pandemics are associated with the introduction of a novel hemagglutinin subtype or a hemagglutinin that is antigenically very different from the circulating epidemic strains. Pandemic outbreaks are unpredictable, and the Mexican flu pandemic in 2009 caused by an H1N1 virus of swine origin certainly took the world by surprise [4]. The impact of H1N1pdm2009 on human health during its first year of circulation was somewhat milder than that caused by most seasonal H3N2 influenza viruses. However, most fatal cases associated with the H1N1pdm2009 virus occurred in young people, which is unusual for seasonal flu [5], [6].

Seasonal influenza vaccines offer little if any protection against a pandemic virus. In 2009, it took six months before a monovalent H1N1pdm2009 vaccine could be distributed: in other words, it became available only after the first wave of the pandemic virus attack [7]. Different approaches are being followed to develop influenza vaccines with broadened immune protection in order to control pandemic influenza outbreaks more efficiently. For example, attempts to introduce antibody-based immunity directed against conserved parts of the hemagglutinin have met this goal with some success [8], [9], [10]. Other universal influenza vaccine candidates are based on the induction of broadly reactive T-cell responses [11], [12], [13]. We and others have focused on the development of a recombinant protein vaccine based on the conserved, extracellular domain of the influenza A matrix protein 2 (M2e) linked to a carrier [14], [15], [16], [17], [18].

M2 is an integral membrane protein of 97 amino acid residues. It is scarce on virus particles but abundant on virus-infected cells [19], [20]. It self-assembles as a homo-tetramer into a proton-selective ion channel [21]. During budding, M2 also changes the cell membrane curvature and promotes membrane scission and hence release of newly formed virions [22]. M2e is the amino-terminal extracellular part of M2 and consists of 23 amino acid residues. It is minimally immunogenic during infection and following conventional vaccination [23]. This might partially explain its remarkable sequence conservation across all human influenza A strains [24]. In addition, the coding information for M2e overlaps with the open reading frame of M1. This imposes a genetic constraint that limits the tolerance for mutations in M2e [25].

We fused M2e to the Hepatitis B virus core capsomer (HBc) to produce recombinant virus-like particles (VLPs). These VLPs, which display M2e on their surface at high density, are highly immunogenic and induce protection against the death and morbidity caused by challenge with influenza A virus [16], [26]. Induction of protection against multiple HA-subtypes of influenza by M2e vaccines has been confirmed by using various carrier conjugates fused chemically or genetically and in both mouse and ferret models [15], [27], [28], [29], [30]. Protection induced by immunization with M2e-fusion constructs against experimental challenge is largely dependent on antibodies directed against M2e, although a contribution of an MHC class-II-restricted CD4^+^ T-cell epitope in M2e is likely also involved [16], [31]. Antibodies directed against M2e lack virus-neutralizing activity. Instead, protection by M2e-specific IgG antibodies relies on Fc receptors and innate immune cells such as macrophages and natural killer cells [32], . Interestingly, and in line with the infection-permissive nature of M2e-based vaccines, exposure of M2e-immune mice to influenza A virus infection is compatible with the induction of cross-reactive T cells [35].

HBc-fusions are frequently used to present weakly immunogenic determinants to the immune system. HBc is a powerful immunogen that functions as both a T-cell-dependent and a T-cell-independent antigen [36] and can induce strong humoral [37], T helper (Th) [38] and even cytotoxic T cell (CTL) responses [39]. The HBc gene can be expressed efficiently in *Escherichia coli*, and VLPs produced in this way are indistinguishable by electron microscopy and physico-chemical analyses from HBc particles produced by virally infected liver cells [40]. In-frame insertions of heterologous epitopes in the HBc subunit at the DNA level usually do not interfere with VLP formation, and these epitopes can be displayed as a dense, highly immunogenic constellation [41]. The HBc capsomer contains a carboxy-terminal nucleic acid binding domain that is important for the encapsidation of the HB virus genome [42]. During recombinant expression and VLP assembly of HBc capsomers in *E. coli*, RNA from the bacterial host is also incorporated within VLPs. This incorporated prokaryotic RNA can act as an adjuvant to prime Th1 immunity directed against the HBc antigen [43].

Here, we compared the immunogenicity and protective efficacy of M2e-VLPs containing or lacking part of the nucleic acid binding domain of HBc. We demonstrate that in the presence of this domain, VLPs with or without the amino-terminal M2e fusion contain nucleic acids derived from the *E. coli* host. In addition, immunization of BALB/c mice with M2e-VLPs containing nucleic acids resulted in a higher M2e-specific serum IgG2a response as well as higher IFNγ responses in splenocytes. Although immunization of mice with M2e-VLPs (whether lacking or containing nucleic acids) protected against a lethal challenge with mouse-adapted X47 (H3N2) virus or PR/8 virus, protection against weight loss was significantly improved by the presence of entrapped RNA. We conclude that nucleic acids entrapped within M2e-VLPs improve protection against the morbidity caused by influenza A virus challenge.

## Materials and Methods

### 2.1. Preparation and Analysis of Recombinant HBc VLPs

**Figure 1:**
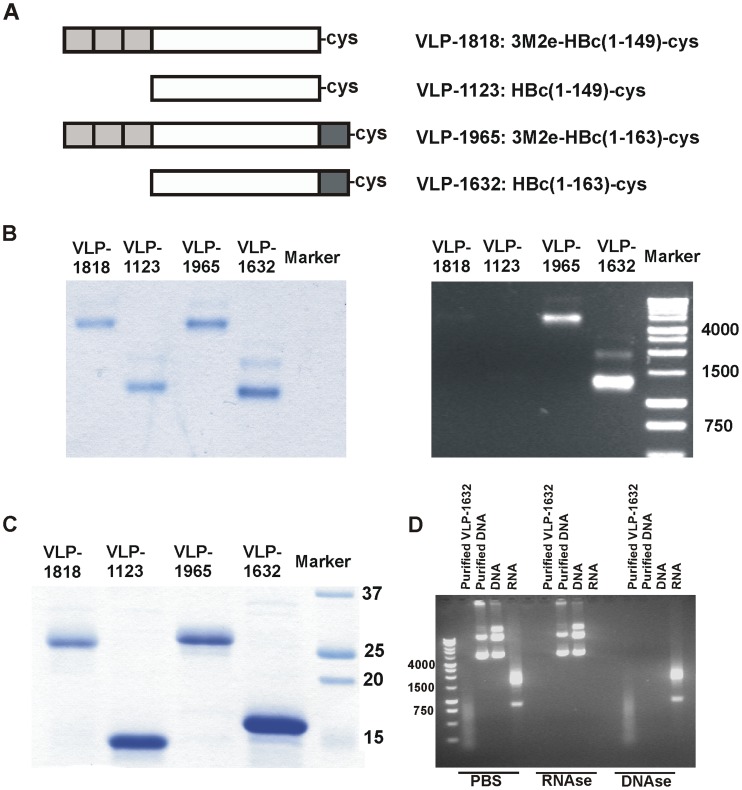
The C-terminal arginine-rich domain of recombinant HBc-based VLPs binds nucleic acids. **A**. Schematic representation of the recombinant M2e-VLPs and control VLPs used in this study. Light grey boxes on the left in VLP-1818 and VLP-1965 represent three tandem M2e copies; white boxes represent HBc amino acid residues 1–149; dark grey boxes on the right in VLP-1965 and VLP-1632 represent part of the arginine-rich domain of HBc (residues 150–163). In all constructs, a C-terminal cysteine residue (cys) was engineered to improve particle stability. **B**. Native agarose gel analysis of VLPs, followed by staining with Coomassie Brilliant blue (left) or ethidium bromide (right). **C**. Purified recombinant VLPs (2 µg each) were separated by SDS-PAGE and stained with Coomassie Brilliant Blue. **D**. VLP-1632 contains RNA. One µg of nucleic acids purified from VLP-1632, similarly purified plasmid DNA, plasmid DNA and total mammalian cellular RNA were treated with either PBS, RNAse or DNAse and analyzed by agarose gel electrophoresis followed by SYBR Safe staining. Numbers on the left indicate sizes in base pairs of the DNA marker in the left hand lane.

All VLPs were produced in and purified from *E. coli*. The VLPs used in this study are shown schematically in Figure 1A. VLP-1123 (M2e−/RNA-) and VLP-1818 (M2e+/RNA-) have been described [44]. VLP-1123 was derived from a truncated HBc gene coding for amino acids 1–149 followed by a cysteine residue. VLP-1818 contains three tandem copies of M2e fused to the N-terminus of VLP-1123. The N-terminal M2e copy in VLP-1818 retains the initiator methionine. In the first and second copy of M2e, the cysteines at positions 17 and 19 are mutated to serines, whereas the third M2e copy retains its cysteines. VLP-1632 (M2e−/RNA+) and VLP-1965 (M2e+/RNA+) are similar to VLP-1123 and VLP-1818, respectively, except that the HBc carrier consists of amino acids 1–163 followed by a cysteine residue. Therefore, VLP-1632 and VLP-1965 contain part of the arginine-rich carboxy-terminal domain of HBc.

All VLPs were expressed from a modified pKK223-3 plasmid (Amersham Pharmacia, UK) [44] and purified as described previously [45]. Briefly, overnight cultures of *E. coli* TB1 transformed with the respective expression vectors were harvested by centrifugation and lysed in Tris-EDTA buffer (50 mM Tris-HCl, 10 mM EDTA, pH 8.0) using a French press (ThermoSpectronic, Cambridge, United Kingdom). Proteins in the cleared lysate were precipitated with ammonium sulfate and the precipitate was resuspended in Tris-EDTA buffer (50 mM Tris-HCl, 10 mM EDTA, pH 8.0). Recombinant particles were purified from the dialyzed ammonium sulfate fraction by gel filtration chromatography on a Sepharose CL-4B column (Pharmacia, Piscataway, N.J.), followed by hydroxyapatite chromatography (Clarkson, South Williamsport, Pa.) and anion-exchange on a Mono-Q (Pharmacia). Purified VLPs were dialyzed against 20 mM sodium phosphate, pH 6.8, and the protein concentration was determined using a standard bicinchoninic acid assay (Pierce). The efficiency of particle formation and the presence of particle-bound nucleic acids were analyzed by agarose gel electrophoresis. VLPs (10 µg) were loaded on a 2% agarose gel and run for 2 h at 80 V in TAE buffer. The gel was stained with ethidium bromide to reveal nucleic acids and with Coomassie brilliant blue to reveal proteins. In addition, 2 µg of each VLP was boiled for 5 min in reducing Laemmli sample buffer, separated by SDS-PAGE, and stained with Coomassie brilliant blue.

Nucleic acids from 40 µg VLP-1632 were isolated using the NucleoSpin RNA Virus kit (Macherey-Nagel) according to manufactures recommendations, including the optional proteinase K treatment but without addition of carrier RNA. Total cellular RNA was isolated from HEK293T cells using by the use of RNeasy kit (Qiagen, Hilden, Germany). One µg of purified VLP-1632 nucleic acids, cellular RNA, miniprep plasmid DNA, as well as control plasmid mini prep DNA that was additionally submitted to the procedures used for the isolation of the VLP nucleic acids were treated with either PBS, 5 µg RNase from bovine pancreas (Sigma-Aldrich, St. Louis, USA) or 1.4 Kunitz of DNAse I (Qiagen) for 30 minutes at 25°C. The samples were then analysed by agarose gel electrophoresis followed by SYBR Safe staining.

The LPS-content of different VLP preparations was determined using the Toxinsensor Chromogenic LAL Endotoxin Assay Kit (GenScript, NJ, USA) according to the manufacturer’s instructions. Briefly, a standard curve was generated from a two-fold dilution series of *E. coli* endotoxin standard in LAL reagent water (1 EU –0.125 EU). Two-fold dilution series of VLPs were made in LAL reagent water and assayed along with the standard and a blank control. After 10 min incubation at 37°C of samples and controls with LAL (Limulus Amebocyte Lysate), and at the end of the reactions absorption was measured spectrophotometrically at 545 nm, using LAL reagent water as a blank. Endotoxin contents in VLP preparation were calculated using the standard curve and expressed as EU/mL or EU/µg of protein.

### 2.2. Mouse Vaccination and Challenge

All mouse experiments were conducted according to the national (Belgian Law 14/08/1986 and 22/12/2003, Belgian Royal Decree 06/04/2010) and European (EU Directives 2010/63/EU, 86/609/EEG) animal regulations. Animal protocols were approved by the ethics committee of Ghent University (permit number LA1400091, approval ID 2010/001). All efforts were made to minimize the suffering of the animals. Specific-pathogen-free female BALB/c mice were obtained from Charles River (France) and immunized at the age of eight weeks. The animals were housed in a temperature-controlled room (biosafety level 2) with 14/10-h light/dark cycles and received food and water *ad libitum.*

M2e-VLPs or control VLPs (10 µg each) were used without adjuvant or adjuvanted with Alhydrogel (Brenntag Biosector A/S Fredrikssund, Denmark) and injected intraperitoneally (i.p.). Particles were adsorbed using a 10% (v/v) solution of a 2.0% Alhydrogel stock solution prior to injection. Mice were primed and boosted at intervals of two or three weeks as indicated in the figure legends. To evaluate the protective efficacy of the vaccine, mice were challenged with 4 LD_50_ of mouse-adapted X47 (H3N2) virus or with 4 LD_50_ of PR/8 virus, as indicated in the figure legends. The mice were anesthetized by i.p. injection of a mixture of ketamine (12 mg/kg) and xylazine (60 mg/kg). Survival and body weight were monitored for 14 days after challenge.

### 2.3. Determination of Serum Antibody Levels and M2e-specific Cellular Responses

Two weeks after each immunization, blood samples were collected from the lateral tail vein. Blood was allowed to clot for 30 min at 37°C, and serum was obtained by combining the supernatants from two successive centrifugations. The titers of M2e- specific antibodies and of IgG subclasses in the serum were determined by ELISA in 96-well Maxisorp immuno-plates (Nunc, Roskilde, Denmark) coated overnight with M2e peptide (2 µg/ml in carbonate buffer, 50 µl/well, 37°C). After coating, plates were washed twice with PBS +0.1% Tween 20 and blocked with 3% milk in PBS. Next, three-fold serial dilutions of mouse serum, starting with a 1/100 dilution, were dispensed in the wells and incubated for 1 h. Sheep-derived anti-mouse serum conjugated with horseradish peroxidase (GE Healthcare UK Ltd, Chalfont St. Giles, U.K.), horseradish peroxidase-conjugated Abs specific for mouse isotypes IgG1 or IgG2a (Southern Biotechnology Associates, Birmingham, AL) and tetramethylbenzidine substrate (Sigma–Aldrich) were used to determine specific antibody titers. Antibody titers are defined as the reciprocal of the highest dilution with an OD_450_ that is at least double the OD_450_ of preimmune serum samples. HBc-specific antibodies were determined using a sandwich ELISA as described [44].

M2e-specific T cell responses were evaluated by measuring interferon-γ (IFN-γ) by enzyme-linked immunospot (ELISPOT). This analysis was done on splenocytes from mice that had been immunized twice two weeks apart by i.p. injection of Alhydrogel-adjuvanted VLP-1123, VLP-1632, VLP-1818, VLP-1965 or PBS. ELISPOT plates were purchased from U-Cytech Biosciences (Utrecht, The Netherlands) and used according to the manufacturer’s protocol. Briefly, 96-well immunoplates were coated with monoclonal anti-IFN-γ antibodies and blocked with blocking buffer. Fourteen days after the second immunization, and six days after challenge with 4 LD_50_ of X47 virus, the spleens of five mice per group were isolated aseptically and single-cell suspensions were prepared. After lysis of red blood cells with ammonium buffer, 3×10^5^ splenocytes were plated in 100 µl of culture medium supplemented with 4 µg/ml of HPLC-purified peptide for restimulation. Splenocytes from each mouse were analyzed in triplicate. The M2e-specific peptide was SLLTEVETPIRNEWGCRCNDSSD (M2e), and the negative control peptide was MNNATFNYTNVNPISHIR. For ELISPOT analysis of splenocytes isolated six days after challenge, nucleoprotein-specific responses were determined by restimulation with the H2d-restricted NP-derived TYQRTRALV peptide. After 16 h of peptide restimulation, plates were washed with ELISA wash buffer and IFN-γ trapped on the plates was detected by a biotinylated polyclonal anti-IFN-γ antiserum. Subsequent incubation with GABA-conjugated streptavidin was used to develop silver spots at places were immune cells secreted IFN-γ during peptide restimulation. Splenocytes from each mouse were analyzed in triplicate and the spots were counted using an inverted light microscope.

### 2.4. Bone-marrow Derived Dendritic Cell (BMDC) Isolation, Stimulation and RT-qPCR Analysis

Bone-marrow was isolated by flushing the tibia and the femur of WT, MyD88^−/−^ and TRIF^−/−^ mice (all in the Bl/6 background) with ice-cold PBS. After lysing red blood cells with ACK red blood cell lysis buffer (BioWhittaker, Wakersville, MD, USA), 3.5 x 10^6^ bone marrow cells were seeded in petri dishes and cultured for 8 days in 10 ml RPMI 1640 (Invitrogen, Merelbeke, Belgium) supplemented with 10% fetal calf serum and 20 ng/ml GM-CSF (Peprotech, Londen, UK). Medium was replenished every 3 days. On day 8, 1 x 10^6^ immature dendritic cells were stimulated with 20 µg of VLP-1123, VLP-1632, VLP-1818, VLP-1965 or 1 µg ODN 1826 (Invivogen, San Diego, CA) for 20 h. RNA was isolated by using the RNeasy Plus Mini Kit (Qiagen, Hilden, Germany) according to the manufacturer’s protocol. cDNA was synthesized using a Superscript II Reverse Transcription Reagent Kit (Invitrogen, Merelbeke, Belgium). Real-time quantitative PCR (qPCR) was performed on a Lightcycler 480 using a qPCR kit for SYBR Green I (both Roche Diagnostics, Mannheim, Germany). Real-time qPCR amplification was performed in triplicate reactions. *mRPL13a* mRNA was used as a reference housekeeping gene for normalization. All primers were purchased from Invitrogen. The PCR conditions were as follows: preincubation at 95°C for 5min; 50 cycles at 95°C for 10s and at 60°C for 30s. The following forward and reverse primers were used: 5′-TAGTCCTTCCTACCCCATTTCC-3′ and 5′-TTGGTCCTTAGCCACTCCTTC-3′ (murine *il6*); 5′-CACCTCACAAGCAGAGCACAAG-3′ and 5′- GCATTAGAAACAGTCCAGCCCATAC-3′ (murine *il1*β); 5′-CCTGCTGCTCTCAAGGTTGTT-3′ and 5′-TGGCTGTCACTGCCTGGTACTT-3′ (murine *rpl13a*).

### 2.5. Determination of Lung Virus Titers

Three to five mice from each group were killed by cervical dislocation four or six days after challenge. The lungs were removed aseptically and homogenized in 1 ml of sterile, ice-cold PBS. The extracts were transferred to centrifuge tubes and cell debris was pelleted for 10 min at 400 g and 4°C. The titers of infectious virus were determined in triplicate by titration of the cleared lung extracts on MDCK cells. Briefly, monolayers of cells were infected for 1 h with 50 µl of serial 1∶10 dilutions of the lung homogenates in a 96-well plate in serum-free DMEM medium containing penicillin and streptomycin (Invitrogen). Following infection, the medium was replaced by medium containing 2 µg/ml of TPCK-treated trypsin (Sigma-Aldrich, Germany). Endpoint virus titers were determined after four days, as described by Reed and Muench, by interpolating the dilution that infected 50% of the wells, as determined by the presence of chicken red blood cell hemagglutinating activity in the supernatants.

### 2.6. Histopathology

Four days after challenge, mice were euthanized by cervical dislocation and samples of the apical, median and caudal regions of the lungs (bilateral) were removed, fixed in 4% paraformaldehyde, embedded in paraffin wax, sectioned at 5 µm, and stained with hematoxylin-eosin. Slides were examined in a blinded fashion using an Olympus BX 50 microscope and micrographs were made.

### 2.7. Statistical Analysis

Comparison of antibody titers between two groups was done with the two-tailed Student’s t-test. Survival rates were plotted as Kaplan-Meier curves and analyzed with the log-rank test. Morbidity parameters after infection of more than two groups were compared using the Holm-Sidak method. Statistical analyses were performed with Graphpad Prism version 4.00 for Windows (GraphPad Software, San Diego California, www.graphpad.com) and with the R language and environment for statistical computing, R Development Core Team, 2009 (R Foundation for Statistical Computing, Vienna, Austria (ISBN 3-900051-07-0, www.R-project.org).

## Results

### *In vitro* Characterization of M2e-VLPs Containing or Lacking Bound Nucleic Acids

Previously, we reported on the characterization and immuno-protective efficacy of M2e-VLPs produced by the HBc presentation technology [16], [44]. In these studies we used HBc VLPs that either contained or lacked the carboxy-terminal arginine-rich domain of HBc. This domain is responsible for nucleic acid binding and was shown to prime the mammalian immune response towards a Th1 response because it facilitates the incorporation of RNA within the HBc-based VLP [43]. Such a Th1 response is favorable for anti-M2e mediated protection [32], [44]. To investigate if HBc VLPs containing the carboxy-terminal arginine-rich domain can induce a more favorable M2e immune response than HBc VLPs lacking this domain, we constructed M2e-presenting HBc VLPs and corresponding control VLPs. The carboxy-terminal arginine-rich domain was either included [VLP-1965 (M2e+/RNA+) and VLP-1632 (M2e−/RNA+)] or excluded [VLP-1818 (M2e+/RNA−) and VLP-1123 (M2e−/RNA−)] (Figure 1A). SDS-PAGE analysis demonstrated that the purified particles were composed of subunits of the expected molecular weight (Figure 1C). In native agarose gel electrophoresis, all VLPs migrated predominantly as single bands, confirming their uniform particulate nature [46] (Figure 1B, left panel). As expected, co-migrating nucleic acids, revealed by ethidium bromide staining, were clearly detectable only in VLP-1965 and in the corresponding control particle VLP-1632, both of which harbour the nucleic acid binding domain (Figure 1B, right panel). Incubation of the particles at 25°C for one hour with RNase or DNase failed to remove the nucleic acids, suggesting that the nucleic acids were trapped within the VLPs and hence protected from exogenous nucleases under these conditions (data not shown). Therefore, we isolated the nucleic acids from VLP-1632 particles by proteinase K treatment followed by silica membrane-based purification. These purified nucleic acids derived from VLP-1632 were susceptible to RNAse and resistant to DNAse treatment (Figure 1D). We conclude that extending the HBc capsid protein at its amino-terminal end with multiple copies of M2e affects neither VLP formation nor the nucleic acid (most likely RNA) binding capacity of these VLPs.

### Nucleic Acids Bound to M2e-VLPs Correlates with a Th1-polarized Humoral Immune Response

**Figure 2:**
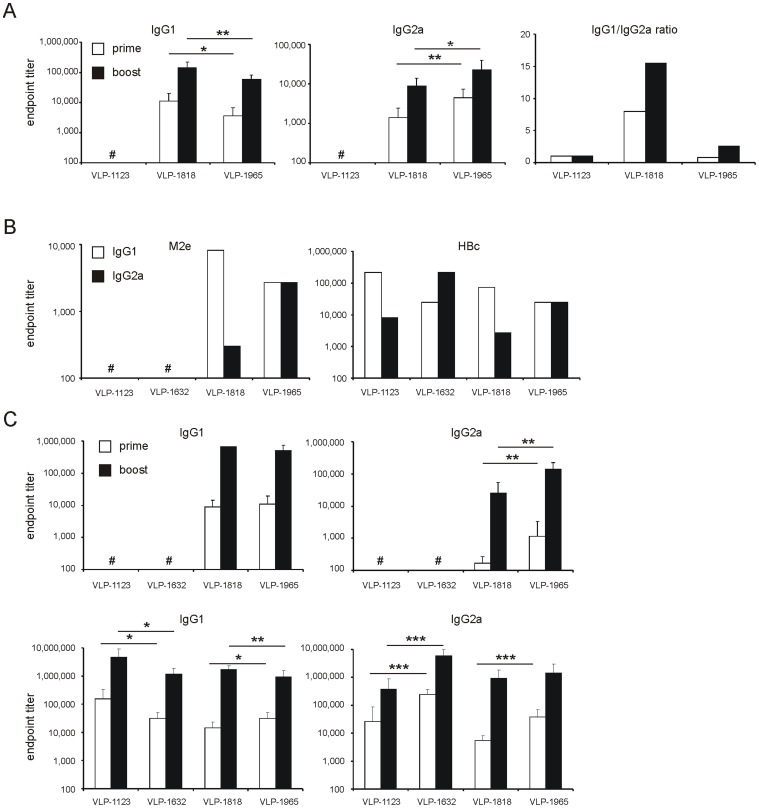
VLPs containing nucleic acids promote a Th1-polarized humoral immune response. **A**. Three groups of 16 BALB/c mice were immunized twice intraperitonealy with 10 µg of VLP-1123, VLP-1818 or VLP-1965, without adjuvant. Priming and boosting injections were given at three week intervals. Two weeks after the boost, M2e-specific serum IgG1 and IgG2a titers were determined by M2e peptide ELISA. White and black bars: serum samples after priming and boosting, respectively. IgG1/IgG2a ratios were calculated by dividing the mean of the IgG1 endpoint titers by the mean of the IgG2a endpoint titers, and are shown on the right. (*: *P*<0.05, **: *P*<0.01 two-sample t-test). **B**. The particles were adjuvanted with Alhydrogel and used to immunize BALB/c mice (14 per group, 10 µg of VLP i.p. per animal). Two weeks after priming, M2e-specific (left) and HBc-specific (right) IgG1 and IgG2a titers were determined in sera pooled from the 14 mice. **C**. Groups of 12 BALB/c mice were immunized twice by i.p. injection of 10 µg of VLP-1123, VLP-1623, VLP-1818 or VLP-1965 formulated with Alhydrogel. Priming and boosting injections were three weeks apart. M2e-specific serum IgG1 and IgG2a titers are shown in the top panels and HBc-specific titers in the bottom panels. White and black bars: serum samples two weeks after priming and boosting, respectively. (*: *P*<0.05, **: *P*<0.01, ***: *P*<0.001, two-sample t-test). Bars in A and C represent averages and error bars represent standard error of the mean. #: titer lower than 100.

We next compared the immunogenicity of the VLP types. BALB/c mice (n = 16 per group) were immunized by i.p. injection of 10 µg of unsubstituted control VLPs, VLP-1818 or VLP-1965. In the absence of added adjuvant, the M2e-specific IgG1 titers in serum were significantly lower in mice that had been immunized with VLP-1965 than in VLP-1818 immunized mice. Conversely, M2e-specific IgG2a titers were significantly higher in mice immunized with VLP-1965 compared to VLP-1818 vaccine recipients, and this was true after both priming and boosting (Figure 2A).

Adjuvants promote the immunogenicity of M2e-fusion constructs [16]. The alum-based adjuvant Alhydrogel is suitable for human use and we previously reported that this adjuvant enhances the immunogenicity of M2e-VLPs carrying a single M2e epitope [44]. Therefore, we also analyzed the effect on immunogenicity of the particle-associated nucleic acids when these particles were formulated with Alhydrogel. To be able to compare our results with those of Riedl *et al.*, who used a single immunization without adjuvant [43], we analyzed M2e- and HBc-specific antibodies two weeks after priming in groups of 14 BALB/c mice. In this experiment, the IgG1 titers were lower for alum-adjuvanted particles containing bound nucleic acids (VLP-1632 and -1965) than for those lacking it (VLP-1123 and -1818). In contrast, serum IgG2a titers against M2e and against the carrier were clearly higher in recipients of alum-adjuvanted VLPs with associated nucleic acids (Figure 2B).

We next performed an independent prime-boost experiment with alum-adjuvanted particles. M2e-specific serum IgG2a responses were higher following vaccination with VLP-1965 than following VLP-1818 (Fig. 2C). Likewise, significantly lower IgG1 and higher serum IgG2a titers directed against the HBc carrier were apparent when nucleic acid-containing VLPs were used, which was observed for the unsubstituted particles (VLP-1123 compared to VLP-1632) after prime and boost and only after the boost immunization for the M2e-displaying particles VLP-1818 and VLP-1965 (Fig. 2C).

**Figure 3:**
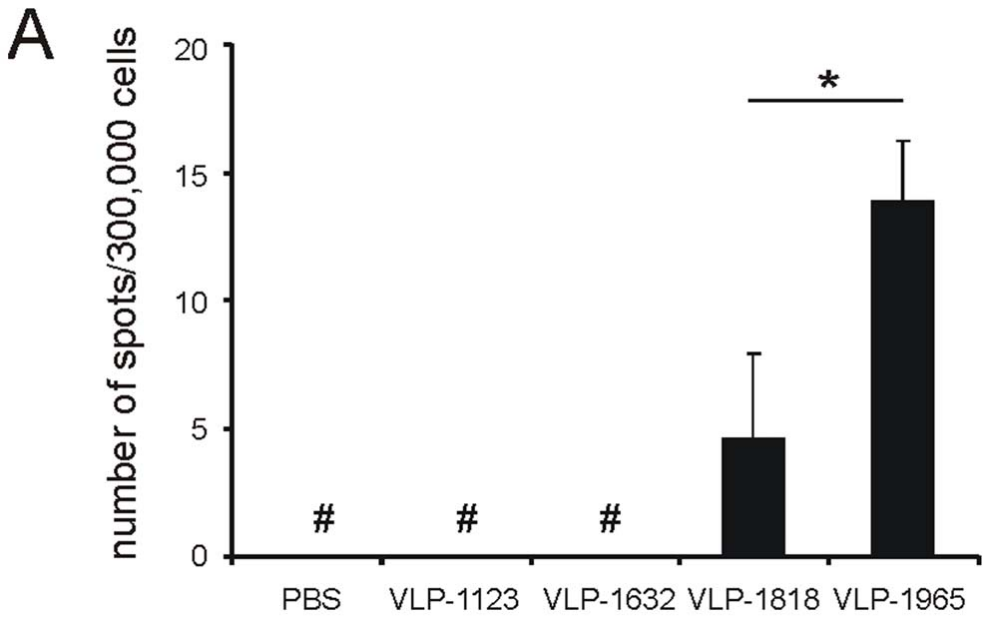
VLPs with nucleic acids promote a Th1-polarized cellular immune response. BALB/c mice (n = 23 per group) were immunized twice by i.p. injection of 10 µg of VLP-1123, VLP-1623, VLP-1818 or VLP-1965 formulated with Alhydrogel. An additional control group received PBS with Alhydrogel (PBS). Priming and boosting injections were two weeks apart. Two weeks after the boost, splenocytes were isolated from five mice in each group and analyzed in triplicate for each mouse. Following 16 h of *in vitro* stimulation of splenocytes with M2e peptide, IFN-γ secreting cells were quantified by ELISPOT analysis. Results are shown as the average number of spots per 300,000 splenocytes. Bars represent averages with error bars depicting the standard error of the mean (*:*P*<0.001 two-sample t-test). #: less than 10 spots.

M2e contains an MHC-II-restricted T cell epitope that can contribute to immune protection in the BALB/c mouse model [31]. Therefore, we used IFN-γ ELISPOT to compare the magnitude of the M2e-specific T helper cell responses. Mice that had been immunized with VLP-1965 displayed a significantly stronger splenic Th1 response upon *in vitro* restimulation with M2e peptide than mice immunized with VLP-1818 recipient mice (Fig. 3).

From these results we conclude that extending VLPs with part of the carboxy-terminal nucleic-acid-binding domain of the HBc capsomer correlates with a Th1 type of immune response in BALB/c mice.

### MYD88 and TRIF are Involved in Gene Induction by HBc-based VLPs

**Figure 4:**
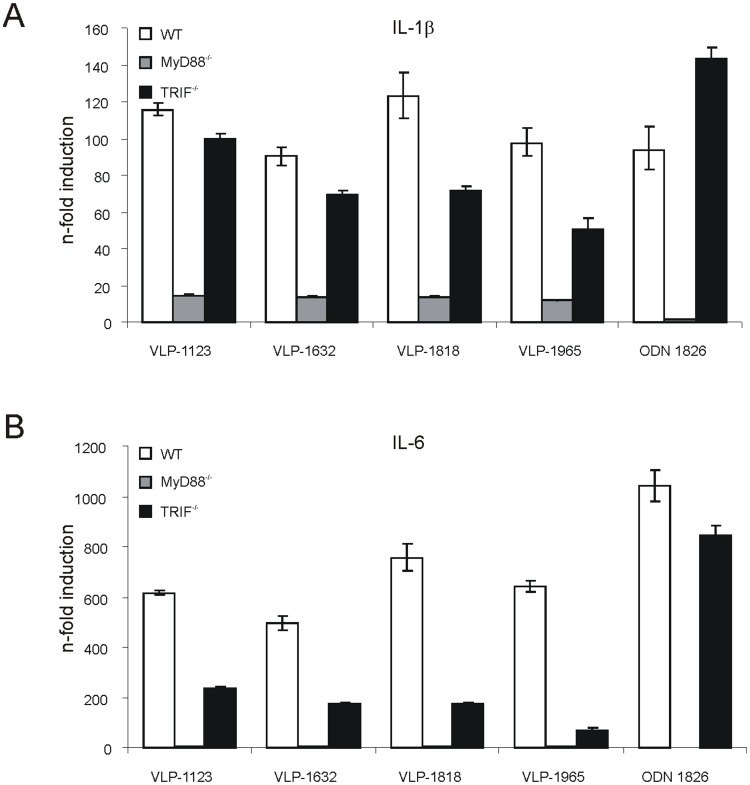
VLP-associated nucleic acids signal through MYD88 and TRIF. RT-qPCR was used to analyze mRNA expression levels of the pro-inflammatory cytokines IL-1β and IL-6 20 hours after stimulation of WT, MyD88^−/−^ or TRIF^−/−^ DCs with 20 µg of VLP-1123, VLP-1632, VLP-1818, VLP-1965 or 1 µg of ODN 1826. Results represent mean n-fold induction levels compared to unstimulated control cells ± SD from triplicate PCR reactions.

Toll-like receptors (TLRs) are vital components of the mammalian innate immune system. They recognize microbial molecular patterns such as double-stranded RNA, 5′ uncapped RNA, CpG DNA, and LPS. Engagement of TLRs by their cognate ligands results in the induction of type I IFN and inflammatory cytokine genes. This TLR-dependent gene activation depends on the adaptor proteins myeloid differentiation primary response protein 88 (MyD88) and TIR domain-containing adapter inducing IFNβ (TRIF) and may promote a subsequent adaptive immune response [47]. To obtain some mechanistic insights in the contribution of these adapter proteins to the immunogenic profile of the VLPs used in this study, we compared the expression levels of the pro-inflammatory genes IL-6 and IL-1β in BMDCs derived from TRIF^−/−^, MyD88^−/−^ and WT mice stimulated with 20 µg of VLP-1123, VLP-1632, VLP-1818 or VLP-1965. As a control, BMDCs were incubated with the TLR-9 agonist ODN 1826 which signals through MyD88. MyD88 deficiency evoked a strong decrease in the expression level of IL-1β and completely blocked the induction of IL-6. In TRIF^−/−^ DCs, expression of IL-6 was profoundly downregulated while the decrease in IL-1β expression was more modest (Fig. 4). Comparison of the LPS content of the *E. coli*-derived recombinant VLPs, revealed a variable level with the lowest amount found in VLP-1123 and VLP-1965 and more significant amounts in VLP-1632 and VLP-1818 (Table 1). Taken together, we found no clear correlation between LPS-content, RNA-content and MyD88- or Trif-dependent gene induction from these *in vitro* BMDC stimulation assays. It is possible that the contribution of the RNA-contained in VLP-1632 and VLP-1965 was masked by LPS or other microbial-derived TLR agonists.

**Table 1:** Characteristics of virus-like particles used in this study.

VLP	Make-up	EU/ml*	EU/µg of protein*
VLP-1123	M2e−/RNA−	2485	2,16
VLP-1632	M2e−/RNA+	50880	46,26
VLP-1818	M2e+/RNA-	250022	320,54
VLP-1965	M2e+/RNA+	7840	18,67

* LPS-content per ml of VLP solution or per µg of protein, expressed as international units and as determined by the LAL-assay.

### Incorporation of Nucleic Acids in VLPs Improves Protection by M2e-VLPs Vaccination

We next compared the protective efficacy of VLP-1818 and VLP-1965 particles. M2e-immune mice survive a lethal influenza A virus challenge but they usually lose some weight. The extent of weight loss in M2e-immune mice depends on the adjuvant, route of immunization and antibody isotype [48]. In BALB/c mice, IgG2a antibodies directed against M2e protect better than their IgG1 counterpart [32]. In independent immunization experiments we consistently noticed significantly higher M2e-specific IgG2a antibody titers in serum of mice that had been primed and boosted with VLP-1965 particles containing nucleic acids than mice that received VLP-1818 (Fig. 2). Because we previously found that immunization with VLP-1818 protected against a potentially lethal challenge with influenza A virus [44], we anticipated that it would require a very large number of mice to obtain a significant difference in survival outcome when applying this parameter as a read out. Therefore, we focused on body weight drop to compare protective efficacy following challenge and we did so in independent experiments. This comparison of efficacy between VLP-1818 and VLP-1965 was important because its outcome would help to guide the choice of the lead M2e-VLP vaccine candidate for a Phase I clinical study.

**Figure 5:**
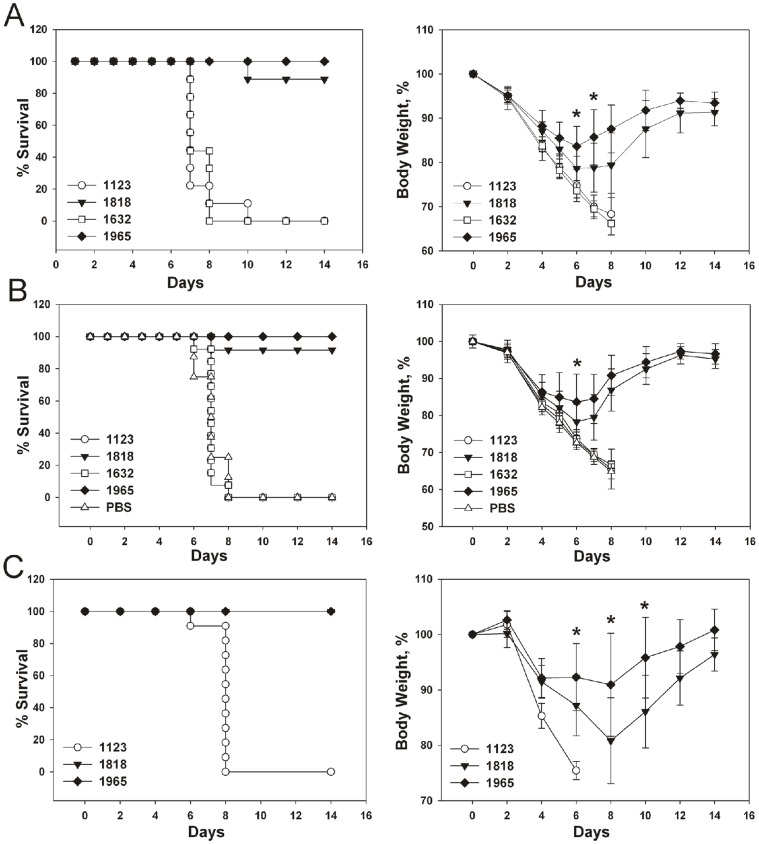
Immunization with VLP-1965 provides better protection against morbidity caused by influenza A virus challenge than VLP-1818. **A.** Groups of 12 BALB/c mice were immunized twice with an interval of three weeks with 10 µg of the indicated VLPs, adjuvanted with Alhydrogel. Three weeks after the second immunization, mice were challenged with 4 LD_50_ of mouse-adapted X47 virus. Survival rate (left; *P*<0.001, Kaplan Meier test) and body weight (right; *P*<0.05, two-sample t-test on days 6 and 7 after challenge) were monitored for two weeks starting from the day of challenge. **B.** Groups of 8 (PBS), 12 (VLP-1818) or 13 (VLP-1965, -1632 and -1123) BALB/c mice were immunized twice with a two-week interval with 10 µg of the indicated VLPs, adjuvanted with Alhydrogel. One group of mice received PBS with Alhydrogel (PBS). Two weeks after the second immunization, mice were challenged with 4 LD_50_ of mouse-adapted X47 virus. Survival rate (left; *P*<0.001, Kaplan Meier test) and body weight (right; *P*<0.05, two-sample t-test on days 6 after challenge) C. Groups of 14 BALB/c mice were immunized three times with three-week intervals by i.p. injection of 10 µg of VLP-1123, VLP-1818 or VLP-1965, adjuvanted with Alhydrogel. Three weeks after the second boost, mice were challenged with 4 LD_50_ of PR/8 virus. Survival rate (left; *P*<0.0001) and body weight (right; *P*<0.05 on days 8, 10 and 12 after challenge).

First, we vaccinated groups of 12 BALB/c mice twice three weeks apart with 10 µg of the VLPs in the presence of Alhydrogel. Three weeks after the boost, mice were challenged with 4 LD_50_ of mouse-adapted X47 virus, and morbidity and mortality were monitored. All mice immunized with VLP-1818 or VLP-1965, except for one in the VLP-1818 group, survived the challenge. Morbidity after challenge was most severe in mice immunized with control VLP-1123 or VLP-1632 and all animals in these groups died after challenge (Figure 5A). Interestingly, during the peak days of morbidity following challenge, VLP-1965-immunized mice lost significantly less body weight than mice immunized with VLP-1818 (Figure 5A).

In an independent second experiment, we compared the protective efficacy in groups of BALB/c mice that had been immunized twice with 10 µg of control or M2e-substituted VLPs. Alhydrogel was used as adjuvant, a PBS-Alhydrogel group was also included and priming and booster immunizations were two weeks apart. Two weeks after the booster immunization, mice were challenged with 4 LD_50_ of mouse-adapted X47 virus and monitored for survival and morbidity. Following challenge, all mice in the control groups gradually lost weight and eventually died or had to be euthanized. In contrast, all VLP-1965 and all except one VLP-1818 immunized mice survived the challenge (Figure 5B). Body weight loss was again higher in the VLP-1818 group and differed significantly from that of the VLP-1965 mice on day 6 after challenge (Figure 5B).

Finally, we also compared the effect of an additional boost vaccination on control of morbidity, between Alhydrogel-adjuvanted VLP-1818 and VLP-1965 vaccines. Groups of 14 mice were used and challenge was with PR/8 virus, which is highly virulent to mice [49]. All VLP-1123 immunized mice died after infection whereas M2e-immunized survived. Again, VLP-1965-immunized mice experienced significantly less body weight loss than mice immunized with VLP-1818 (Figure 5C). In summary, mice vaccinated with VLP-1965 or VLP-1818 survived a potentially lethal influenza A virus challenge. In addition, VLP-1965 was more protective than VLP-1818 against morbidity caused by challenge.

### Lung Virus Load is Reduced in M2e-immune Mice

**Figure 6:**
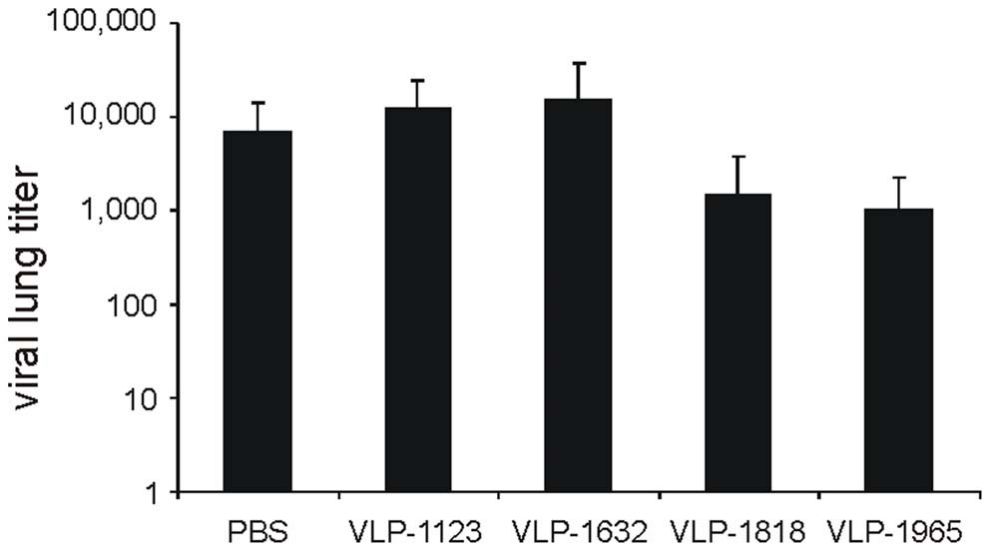
Immunization with M2e-VLPs reduces lung virus replication. BALB/c mice were immunized twice two weeks apart with 10 µg of Alhydrogel-adjuvanted PBS, VLP-1123, VLP-1632, VLP-1818 or VLP-1965. Two weeks after the boost, mice were challenged with 4 LD_50_ of X47 virus. Six days after challenge, four (PBS) or five (all other groups) mice from each group were sacrificed to determine lung virus titers. Virus titers are expressed as mean log_10_ TCID_50_/ml and error bars depict the standard error of the mean.

In the aforementioned challenge experiments, we also determined the lung virus load. Three to five mice from each group were sacrificed on day 4 or 6 after challenge. We consistently observed a one to two log lower virus load in VLP-1818 and VLP-1965 immunized mice compared to control groups. As an example, Figure 6 depicts the lung virus loads in mice that had been immunized twice with 10 µg of Alhydrogel-adjuvanted control (PBS, VLP-1123 or VLP-1632) or M2e-substituted VLPs (VLP-1818 or VLP-1965) and challenged with 4 LD_50_ of X47 virus. However, there was no significant difference in lung virus titer between mice vaccinated with VLP-1965 or with VLP-1818.

### Cellular Immune Responses Following Challenge

**Figure 7:**
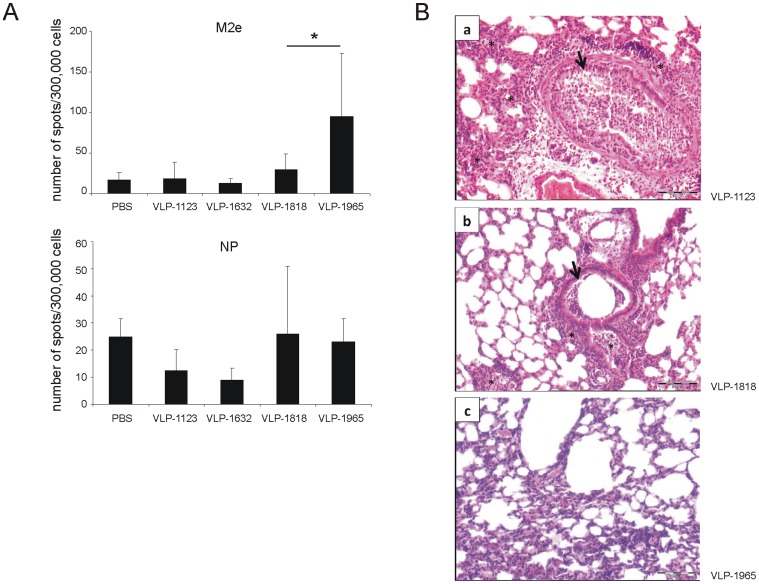
Cellular immune responses and histopathology following challenge. Mice were immunized as described in the legend of Figure 6. **A.** Six days after challenging the mice with 4 LD_50_ of X47 virus, four (PBS) or five (all other groups) mice from each group were sacrificed to prepare splenocytes. IFN-γ responses after M2e (top) or NP (bottom) peptide restimulation were determined by ELISPOT analysis. Bars represent the average number of spots determined for individual mice (analyzed in triplicate) from each group. Error bars represent standard error of the mean. (*:*P*<0.05). **B.** Representative hematoxylin-eosin stained lung tissue section prepared on day four after challenge of mice immunized with 10 µg of Alhydrogel-adjuvanted VLP-1632, VLP-1818 or VLP-1965. VLP-1123 (a): diffuse presence of a pronounced interstitial, alveolar (*) and bronchiolar (arrow) mixed inflammatory infiltrate interlaced with necrotic debris. VLP-1818 (b): focal presence of a limited mixed inflammatory infiltrate both in the interstitial, alveolar (*) as well as bronchiolar (arrow) structures. VLP-1965 (c): Some congestion, but no inflammatory reaction.

We recently reported that M2e-immune mice that are challenged with a sublethal dose of X47 virus mount cellular immune responses against the viral nucleoprotein (NP). These NP-specific T-cell responses were similar in magnitude to those in challenged naïve mice [35]. Therefore, we determined the M2e- and NP-specific T cell responses in splenocytes isolated six days after X47 challenge. An IFN-γ specific ELISPOT analysis of splenocytes stimulated *in vitro* with M2e peptide showed that after challenge, VLP-1965 immunized mice had significantly stronger cellular responses than VLP-1818 immunized mice (Fig. 7A). These responses were presumable due to the activation of CD4^+^ T-cells [31]. However, this result should be interpreted with care because these M2e-specific T cell responses include those induced by vaccination in the VLP-1818 and VLP-1965 immunized mice as well as those induced by the influenza A virus challenge. IFN-γ specific ELISPOT analysis revealed comparable NP-specific responses in splenocytes from all mice, even though M2e-immune mice were protected from challenge whereas control mice died (Figure 7A).

## Discussion

We describe the use of VLP-1965, an improved M2e-based universal influenza A vaccine candidate, and compare its properties and efficacy to the previously reported VLP-1818 [44]. The carboxy-terminus of VLP-1965 comprises part of the arginine-rich domain of HBc (amino acid residues 150–163), allowing these particles to trap nucleic acids. Immunization with either of these M2e-displaying VLPs protected mice from death from a potentially lethal influenza A virus challenge. Furthermore, after challenge, M2e-VLP immunized mice displayed less histopathological abnormalities than control-VLP-immunized mice: the alveolar structure was less affected, and alveolar pathology was more focal (Figure 7B). However, immunization with M2e-displaying VLPs harboring the RNA-binding domain (VLP-1965) protected better against weight loss following challenge than immunization with VLPs lacking this domain (VLP-1818). In contrast, immunization with the control VLPs did not protect and did not result in a difference in morbidity in mice immunized with either control VLP. This indicates that the reduction in morbidity was due to induction of an adaptive immune response against the M2e-antigen and not to the induction of inflammation. Lung virus titers were lower in M2e-immunized mice compared to the controls whereas viral loads determined on day six after challenge were comparable in the VLP-1818 and VLP-1965 immunized mice. This may be explained by the lack of in vitro neutralizing activity of M2e-specific IgG [35]. In addition, a difference in morbidity does not necessarily correlate with a difference in lung virus load in the mouse model for influenza [50].

M2e-based protection against influenza can be mediated by M2e-specific antibodies but might also depend on M2e-specific CD4 T cells [31]. M2e-specific IgG protects mice from influenza infections by a mechanism that involves both Fcγ receptors and alveolar macrophages [32]. As M2e-specific antibodies probably cannot readily bind to influenza particles, it is likely that M2e-specific antibodies protect against infection by eliminating the infected cells *via* ADCC or phagocytosis. We previously demonstrated that M2e-specific IgG2a antibodies can protect against influenza infections more efficiently than M2e-specific IgG1 antibodies. In addition, M2e-specific IgG1 failed to protect Fcγ receptor III knockout mice, but M2e-specific IgG2a could rescue protection in these animals [32]. Vaccination with M2e-VLPs harbouring the RNA-binding domain induces higher levels of M2e-specific IgG2a antibodies. In this way, the enhanced protection triggered by immunization with M2e-VLPs harboring an RNA-binding domain might be explained by the enhanced induction of IgG2a antibodies. So by promoting the induction of M2e-specific IgG2a antibodies, VLP-1965 could promote Fcγ-receptor-mediated elimination of infected cells, thereby further limiting inflammation and morbidity.

Following immunization, nucleic acids bound within VLP-1965 are presumably taken up by antigen-presenting cells. The vertebrate innate immune system is armed with both extracellular and intracellular molecular sensors that recognize pathogen-associated molecular patterns such as unmethylated CpG DNA and single- and double-stranded RNA [51]. Triggering of these sensors, *e.g.* the Toll-like receptors TLR-3, -7, -8 and -9 by nucleic acids induces a signaling cascade that leads to the expression of several cytokines. These in turn activate the innate immune system, which then programs a specific immune response. Riedl *et al*. demonstrated that deleting the Arg-rich C-terminus of HBc results in particles of 149-aa that retain less than 2% of the RNA binding capacity of full-length HBc [43]. This reduced RNA binding correlated with an IgG-isotype switch from the induction of a predominantly Th1 response with high IgG2a titers (upon vaccination with full-length HBc) towards a Th2 response with high HBc-specific IgG1 antibody titers (upon vaccination with truncated HBc-149 lacking nucleic acids) [43]. VLP-1965 combines three tandem M2e repeats presented at high density on its surface and entraps nucleic acids. Most likely, these nucleic acids (presumably bacterial RNA) bias the M2e-specific immune response towards a Th1-type, as evidenced by the enhancement of the serum titer of anti-M2e IgG2a. A major Th1-type cytokine is IFN-γ. Indeed, based on our ELISPOT data, M2e-specific T cell responses resulting in IFN-γ production were increased in the spleen of VLP-1965 immunized mice. In addition, we compared IL-6 and IL-1β mRNA levels induced by *in vitro* stimulation of differentiated dendritic cells derived from TRIF^−/−^, MyD88^−/−^ and WT mice with VLP-1632 or VLP-1965. In the absence of MyD88 and to a lesser extent of TRIF, the induction of these pro-inflammatory genes was strongly diminished, suggesting the involvement of Toll-like receptors in costimulation. Interestingly, bacterial mRNA co-administered with bacterial antigens, acts as a pathogen-associated molecular pattern that promotes humoral responses, including switching of antibody isotype classes [52].

M2e-specific IgG2a antibodies play an important role in the protection induced by the M2e-vaccine against influenza [32], [46]. These findings are corroborated by the improved protection obtained with VLP-1965 relative to VLP-1818. In addition, we recently demonstrated that M2e-specific IgG1 failed to protect Fcγ-receptor III knockout mice, and that M2e-specific IgG2a could rescue protection in these animals [32]. An important role for IgG2a antibodies in protection against influenza has also been demonstrated in SCID mice by passive immunization with anti-M2e IgG monoclonal antibodies [32], [53].

Conventional influenza vaccines hamper the development of heterosubtypic immunity endowed by T-cells [35]. Such T cell responses are induced by natural infection with influenza viruses and many of these responses are cross-protective because they are mainly directed against conserved internal structural proteins. Following infection with influenza A virus, M2e-immune mice mount cytotoxic T cell responses directed against NP and HA that are comparable to those in sham-vaccinated mice, as we recently reported in a study involving the VLP-1818 vaccine antigen [35]. It was beyond the scope of the current work to extensively compare such T cell responses in challenged mice that had either been vaccinated with either VLP-1818 or the Th1-biasing VLP-1965 vaccine, but nevertheless we found that the latter particles did not compromise the T cell responses directed against NP (Figure 7).

VLP-1965 was recently evaluated successfully in a Phase I clinical study to document its safety and immunogenicity in humans (ClinicalTrials.gov, Identifier NCT00819013). The M2e-vaccine remains a valid pandemic influenza vaccine candidate and also holds promise as a vaccine that could be used in young children to control disease without compromising T cell immunity upon exposure to seasonal or pandemic influenza [35]. Alternatively, the M2e-vaccine could be used as an adjunct of the currently licensed influenza vaccines to broaden their protective efficacy [34]. VLP-1965 is a promising candidate for such strategies.

## References

[1] Thompson WW, Comanor L, Shay DK (2006) Epidemiology of seasonal influenza: use of surveillance data and statistical models to estimate the burden of disease. The Journal of infectious diseases 194 Suppl 2: S82–91. 17163394 10.1086/507558

[2] Wood JS, Robertson JS (2007) Reference viruses for seasonal and pandemic influenza vaccine preparation. Influenza Other Respi Viruses 1: 5–9. 10.1111/j.1750-2659.2006.00003.x PMC4634662 19459278

[3] Nichol KL, Treanor JJ (2006) Vaccines for seasonal and pandemic influenza. J Infect Dis 194 Suppl 2: S111–118. 17163383 10.1086/507544

[4] Garten RJ, Davis CT, Russell CA, Shu B, Lindstrom S, et al (2009) Antigenic and genetic characteristics of swine-origin 2009 A(H1N1) influenza viruses circulating in humans. Science 325: 197–201. 19465683 10.1126/science.1176225 PMC3250984

[5] Dawood FS, Iuliano AD, Reed C, Meltzer MI, Shay DK, et al. (2012) Estimated global mortality associated with the first 12 months of 2009 pandemic influenza A H1N1 virus circulation: a modelling study. The Lancet infectious diseases. 10.1016/S1473-3099(12)70121-4 22738893

[6] Wijngaard CC, Asten L, Koopmans MP, Pelt W, Nagelkerke NJ, et al (2012) Comparing pandemic to seasonal influenza mortality: moderate impact overall but high mortality in young children. PloS one 7: e31197. 22319616 10.1371/journal.pone.0031197 PMC3272034

[7] Robertson JS, Nicolson C, Harvey R, Johnson R, Major D, et al (2011) The development of vaccine viruses against pandemic A(H1N1) influenza. Vaccine 29: 1836–1843. 21199698 10.1016/j.vaccine.2010.12.044

[8] Giles BM, Crevar CJ, Carter DM, Bissel SJ, Schultz-Cherry S, et al (2012) A computationally optimized hemagglutinin virus-like particle vaccine elicits broadly reactive antibodies that protect nonhuman primates from H5N1 infection. The Journal of infectious diseases 205: 1562–1570. 22448011 10.1093/infdis/jis232 PMC3415819

[9] Steel J, Lowen AC, Wang TT, Yondola M, Gao Q, et al. (2010) Influenza virus vaccine based on the conserved hemagglutinin stalk domain. mBio 1. 10.1128/mBio.00018-10 PMC2912658 20689752

[10] Wang TT, Tan GS, Hai R, Pica N, Ngai L, et al (2010) Vaccination with a synthetic peptide from the influenza virus hemagglutinin provides protection against distinct viral subtypes. Proc Natl Acad Sci U S A 107: 18979–18984. 20956293 10.1073/pnas.1013387107 PMC2973924

[11] Berthoud TK, Hamill M, Lillie PJ, Hwenda L, Collins KA, et al (2011) Potent CD8+ T-cell immunogenicity in humans of a novel heterosubtypic influenza A vaccine, MVA-NP+M1. Clinical infectious diseases 52: 1–7. 21148512 10.1093/cid/ciq015 PMC3060888

[12] Hillaire ML, Osterhaus AD, Rimmelzwaan GF (2011) Induction of virus-specific cytotoxic T lymphocytes as a basis for the development of broadly protective influenza vaccines. Journal of biomedicine & biotechnology 2011: 939860. 22007149 10.1155/2011/939860 PMC3189652

[13] Price GE, Soboleski MR, Lo CY, Misplon JA, Quirion MR, et al (2010) Single-dose mucosal immunization with a candidate universal influenza vaccine provides rapid protection from virulent H5N1, H3N2 and H1N1 viruses. PloS one 5: e13162. 20976273 10.1371/journal.pone.0013162 PMC2953831

[14] Bessa J, Schmitz N, Hinton HJ, Schwarz K, Jegerlehner A, et al (2008) Efficient induction of mucosal and systemic immune responses by virus-like particles administered intranasally: implications for vaccine design. European journal of immunology 38: 114–126. 18081037 10.1002/eji.200636959

[15] Mozdzanowska K, Feng J, Eid M, Kragol G, Cudic M, et al (2003) Induction of influenza type A virus-specific resistance by immunization of mice with a synthetic multiple antigenic peptide vaccine that contains ectodomains of matrix protein 2. Vaccine 21: 2616–2626. 12744898 10.1016/s0264-410x(03)00040-9

[16] Neirynck S, Deroo T, Saelens X, Vanlandschoot P, Jou WM, et al (1999) A universal influenza A vaccine based on the extracellular domain of the M2 protein. Nat Med 5: 1157–1163. 10502819 10.1038/13484

[17] Turley CB, Rupp RE, Johnson C, Taylor DN, Wolfson J, et al (2011) Safety and immunogenicity of a recombinant M2e-flagellin influenza vaccine (STF2.4xM2e) in healthy adults. Vaccine 29: 5145–5152. 21624416 10.1016/j.vaccine.2011.05.041

[18] Wolf AI, Mozdzanowska K, Williams KL, Singer D, Richter M, et al (2012) Vaccination with M2e-based multiple antigenic peptides: characterization of the B cell response and protection efficacy in inbred and outbred mice. PloS one 6: e28445. 10.1371/journal.pone.0028445 PMC3236751 22180783

[19] Lamb RA, Zebedee SL, Richardson CD (1985) Influenza virus M2 protein is an integral membrane protein expressed on the infected-cell surface. Cell 40: 627–633. 3882238 10.1016/0092-8674(85)90211-9

[20] Zebedee SL, Lamb RA (1988) Influenza A virus M2 protein: monoclonal antibody restriction of virus growth and detection of M2 in virions. Journal of virology 62: 2762–2772. 2455818 10.1128/jvi.62.8.2762-2772.1988 PMC253710

[21] Sakaguchi T, Tu Q, Pinto LH, Lamb RA (1997) The active oligomeric state of the minimalistic influenza virus M2 ion channel is a tetramer. Proc Natl Acad Sci U S A 94: 5000–5005. 9144179 10.1073/pnas.94.10.5000 PMC24620

[22] Rossman JS, Jing X, Leser GP, Lamb RA (2010) Influenza virus M2 protein mediates ESCRT-independent membrane scission. Cell 142: 902–913. 20850012 10.1016/j.cell.2010.08.029 PMC3059587

[23] Feng J, Zhang M, Mozdzanowska K, Zharikova D, Hoff H, et al (2006) Influenza A virus infection engenders a poor antibody response against the ectodomain of matrix protein 2. Virol J 3: 102. 17150104 10.1186/1743-422X-3-102 PMC1702354

[24] Ito T, Gorman OT, Kawaoka Y, Bean WJ, Webster RG (1991) Evolutionary analysis of the influenza A virus M gene with comparison of the M1 and M2 proteins. J Virol 65: 5491–5498. 1895397 10.1128/jvi.65.10.5491-5498.1991 PMC249043

[25] Lamb RA, Lai CJ, Choppin PW (1981) Sequences of mRNAs derived from genome RNA segment 7 of influenza virus: colinear and interrupted mRNAs code for overlapping proteins. Proc Natl Acad Sci U S A 78: 4170–4174. 6945577 10.1073/pnas.78.7.4170 PMC319750

[26] De Filette M, Fiers W, Martens W, Birkett A, Ramne A, et al (2006) Improved design and intranasal delivery of an M2e-based human influenza A vaccine. Vaccine 24: 6597–6601. 16814430 10.1016/j.vaccine.2006.05.082

[27] Fan J, Liang X, Horton MS, Perry HC, Citron MP, et al (2004) Preclinical study of influenza virus A M2 peptide conjugate vaccines in mice, ferrets, and rhesus monkeys. Vaccine 22: 2993–3003. 15297047 10.1016/j.vaccine.2004.02.021

[28] Roose K, Fiers W, Saelens X (2009) Pandemic preparedness: toward a universal influenza vaccine. Drug news & perspectives 22: 80–92. 19330167 10.1358/dnp.2009.22.2.1334451

[29] Schotsaert M, De Filette M, Fiers W, Saelens X (2009) Universal M2 ectodomain-based influenza A vaccines: preclinical and clinical developments. Expert review of vaccines 8: 499–508. 19348565 10.1586/erv.09.6 PMC2706389

[30] Tompkins SM, Zhao ZS, Lo CY, Misplon JA, Liu T, et al (2007) Matrix protein 2 vaccination and protection against influenza viruses, including subtype H5N1. Emerg Infect Dis 13: 426–435. 17552096 10.3201/eid1303.061125 PMC2725899

[31] Eliasson DG, El Bakkouri K, Schon K, Ramne A, Festjens E, et al (2008) CTA1-M2e-DD: a novel mucosal adjuvant targeted influenza vaccine. Vaccine 26: 1243–1252. 18243429 10.1016/j.vaccine.2007.12.027

[32] El Bakkouri K, Descamps F, De Filette M, Smet A, Festjens E, et al (2010) Universal vaccine based on ectodomain of matrix protein 2 of influenza A: Fc receptors and alveolar macrophages mediate protection. Journal of immunology 186: 1022–1031. 10.4049/jimmunol.0902147 21169548

[33] Jegerlehner A, Schmitz N, Storni T, Bachmann MF (2004) Influenza A vaccine based on the extracellular domain of M2: weak protection mediated via antibody-dependent NK cell activity. Journal of immunology 172: 5598–5605. 10.4049/jimmunol.172.9.5598 15100303

[34] Song JM, Van Rooijen N, Bozja J, Compans RW, Kang SM (2011) Vaccination inducing broad and improved cross protection against multiple subtypes of influenza A virus. Proc Natl Acad Sci U S A 108: 757–761. 21187388 10.1073/pnas.1012199108 PMC3021063

[35] Schotsaert M, Ysenbaert T, Neyt K, Ibanez LI, Bogaert P, et al. (2012) Natural and long-lasting cellular immune responses against influenza in the M2e-immune host. Mucosal immunology. 10.1038/mi.2012.69 22806098

[36] Milich DR, McLachlan A (1986) The nucleocapsid of hepatitis B virus is both a T-cell-independent and a T-cell-dependent antigen. Science 234: 1398–1401. 3491425 10.1126/science.3491425

[37] Salfeld J, Pfaff E, Noah M, Schaller H (1989) Antigenic determinants and functional domains in core antigen and e antigen from hepatitis B virus. J Virol 63: 798–808. 2463383 10.1128/jvi.63.2.798-808.1989 PMC247753

[38] Ferrari C, Bertoletti A, Penna A, Cavalli A, Valli A, et al (1991) Identification of immunodominant T cell epitopes of the hepatitis B virus nucleocapsid antigen. J Clin Invest 88: 214–222. 1711541 10.1172/JCI115280 PMC296022

[39] Milich DR, McLachlan A, Stahl S, Wingfield P, Thornton GB, et al (1988) Comparative immunogenicity of hepatitis B virus core and E antigens. J Immunol 141: 3617–3624. 2460543

[40] Crowther RA, Kiselev NA, Bottcher B, Berriman JA, Borisova GP, et al (1994) Three-dimensional structure of hepatitis B virus core particles determined by electron cryomicroscopy. Cell 77: 943–950. 8004680 10.1016/0092-8674(94)90142-2

[41] Ulrich R, Nassal M, Meisel H, Kruger DH (1998) Core particles of hepatitis B virus as carrier for foreign epitopes. Adv Virus Res 50: 141–182. 9520999 10.1016/s0065-3527(08)60808-8

[42] Hatton T, Zhou S, Standring DN (1992) RNA- and DNA-binding activities in hepatitis B virus capsid protein: a model for their roles in viral replication. J Virol 66: 5232–5241. 1501273 10.1128/jvi.66.9.5232-5241.1992 PMC289076

[43] Riedl P, Stober D, Oehninger C, Melber K, Reimann J, et al (2002) Priming Th1 immunity to viral core particles is facilitated by trace amounts of RNA bound to its arginine-rich domain. J Immunol 168: 4951–4959. 11994446 10.4049/jimmunol.168.10.4951

[44] De Filette M, Min Jou W, Birkett A, Lyons K, Schultz B, et al (2005) Universal influenza A vaccine: optimization of M2-based constructs. Virology 337: 149–161. 15914228 10.1016/j.virol.2005.04.004

[45] Birkett A, Lyons K, Schmidt A, Boyd D, Oliveira GA, et al (2002) A modified hepatitis B virus core particle containing multiple epitopes of the Plasmodium falciparum circumsporozoite protein provides a highly immunogenic malaria vaccine in preclinical analyses in rodent and primate hosts. Infect Immun 70: 6860–6870. 12438363 10.1128/IAI.70.12.6860-6870.2002 PMC133050

[46] Birnbaum F, Nassal M (1990) Hepatitis B virus nucleocapsid assembly: primary structure requirements in the core protein. J Virol 64: 3319–3330. 2191149 10.1128/jvi.64.7.3319-3330.1990 PMC249568

[47] Kawai T, Akira S (2006) TLR signaling. Cell death and differentiation 13: 816–825. 16410796 10.1038/sj.cdd.4401850

[48] Mozdzanowska K, Zharikova D, Cudic M, Otvos L, Gerhard W (2007) Roles of adjuvant and route of vaccination in antibody response and protection engendered by a synthetic matrix protein 2-based influenza A virus vaccine in the mouse. Virology journal 4: 118. 17974006 10.1186/1743-422X-4-118 PMC2186315

[49] Tate MD, Schilter HC, Brooks AG, Reading PC (2011) Responses of mouse airway epithelial cells and alveolar macrophages to virulent and avirulent strains of influenza A virus. Viral immunology 24: 77–88. 21449718 10.1089/vim.2010.0118

[50] Jagger BW, Wise HM, Kash JC, Walters KA, Wills NM, et al (2012) An overlapping protein-coding region in influenza A virus segment 3 modulates the host response. Science 337: 199–204. 22745253 10.1126/science.1222213 PMC3552242

[51] Barbalat R, Ewald SE, Mouchess ML, Barton GM (2011) Nucleic acid recognition by the innate immune system. Annual review of immunology 29: 185–214. 10.1146/annurev-immunol-031210-101340 21219183

[52] Sander LE, Davis MJ, Boekschoten MV, Amsen D, Dascher CC, et al (2011) Detection of prokaryotic mRNA signifies microbial viability and promotes immunity. Nature 474: 385–389. 21602824 10.1038/nature10072 PMC3289942

[53] Mozdzanowska K, Maiese K, Furchner M, Gerhard W (1999) Treatment of influenza virus-infected SCID mice with nonneutralizing antibodies specific for the transmembrane proteins matrix 2 and neuraminidase reduces the pulmonary virus titer but fails to clear the infection. Virology 254: 138–146. 9927581 10.1006/viro.1998.9534

